# The role of magnetic resonance imaging and computed tomography in oral squamous cell carcinoma patients’ preoperative staging

**DOI:** 10.3389/fonc.2023.972042

**Published:** 2023-03-07

**Authors:** Philipp Thoenissen, Anja Heselich, Iris Burck, Robert Sader, Thomas Vogl, Shahram Ghanaati

**Affiliations:** ^1^ Department of Oral, Cranio-Maxillofacial and Plastic Facial Surgery, Goethe University Frankfurt, Frankfurt am Main, Germany; ^2^ Department of Diagnostic and Interventional Radiology, Goethe University Frankfurt, Frankfurt am Main, Germany

**Keywords:** CMF surgery, oncologic surgery, oral squamous cell carcinoma (OSCC), staging, CT, MRI, ND

## Abstract

**Introduction:**

The aim of the study was to evaluate the accuracy of MRI and CT with regard to the detection of lymph node metastases based on the data of specific patients with OSCC who received bilateral neck dissection.

**Materials and methods:**

In a retrospective analysis from 01/2014 to 12/2020 patients who underwent primary tumor resection and bilateral neck dissection were evaluated.

**Results:**

174 preoperative MRI (78.74%, N=137) and CT (21.26%, N=37) were correlated with the histopathological findings. CT had a sensitivity of 67% and specificity of 68% (p=0.76). MRI showed an overall sensitivity of 66% and a specificity of 68% (p=0.76). In 52.87% of all cases no differences between cN and pN were found. MRI is the method to overestimate lymph node involvement compared to CT (overestimation in 27% vs. 21.62%).

**Conclusion:**

The current data indicate that MR and CT show poor efficacy in the detection of cervical metastases. Accordingly, attention must be paid to alternatives to correct local staging modalities. The application of structured bilateral neck dissection needs to be questioned.

## Introduction

Oral squamous cell carcinoma (OSCC) remains a common and life-threatening disease despite advances in the imaging modalities and therapeutic treatment. At the time of initial diagnosis, local cervical lymph node metastasis is diagnosed in 23 to 35% of all patients, and neck dissection (ND) contributes significantly to the overall survival and oncologic outcome of patients (1–3).

Lymph node involvement, tumor size and systemic metastases are the basis for the discussion of a patient’s case in a multidisciplinary oncology board meeting and are thereby predetermined by imaging and established therapy modalities. Hence, detailed information on each patients’ status is of the utmost importance. Standard imaging technologies currently employed include ultrasound, magnetic resonance imaging (MRI), computed tomography (CT) and positron emission tomography (PET)-CT (4, 5).

These diagnostic modalities complement the clinical examination and primarily influence the extent of lymph node removal. This information determines the final presurgical tumor staging according to cTxNxMx. Therapy consists of primary surgery, radiotherapy or a combination of both, often combined with chemotherapy/immunotherapy. Standard surgical therapy includes tumor resection and elective or therapeutic lymph node dissection, either unilateral or bilateral, in combination with defect closure. However, due to the current lack of a wide variety of studies and guidelines worldwide, there is still no standard in performing neck dissection in patients with OSCC concerning extent to site or level according to Robbins or number of nodes dissected (6).

Generally, a distinction is made in ND between elective and therapeutic procedures. An elective ND is performed if there is no clinical evidence of lymph node involvement. A therapeutic ND is conducted if there is clinical evidence of lymph node involvement at initial diagnosis or in case of tumor recurrence. The latter is also referred to as salvage ND (3). This classification is independent of the extent of ND. The extent of ND is determined by anatomical regions introduced by *Robbins (1991/2002)* as a general classification on the topography of 6 levels of cervical lymph nodes. The levels are defined within anatomical structures of the head and neck region (see **Table 1**) (6, 7). According to this classification, 4 types of ND have been described: radical neck dissection (RND), modified radical neck dissection (MRND), selective neck dissection (SND), and extended neck dissection (END) (6, 7). RND is the removal of lymphatic tissue to level V, including the spinal accessory nerve (SAN), the internal jugular veine (IJF) and the sternocleidoid muscle (SCM). The MRND spares one or more of these structures. *Medina* (1989) proposes subclassifications for MRND: MRND I preserves SAN; MRND II SAN and IJF; and MRND III SAN, IJF, and SCM; within this classification, type A comprises levels I to V, while type B comprises levels II-V (8) (**Table 2**).

**Table 1:** Levels and their boundaries according to the Robbins classification.

Level	Name and boundaries
**Level IA**	Submental (anterior: symphysis, inferior: hyoid, medial: anterior belly of contralateral digastric muscle, lateral: anterior belly of ipsilateral digastric muscle)
**Level IB**	Submandibular (mandible; posterior belly of muscle; anterior belly of digastric muscle; stylohyoid muscle)
**Level II A and B**	Upper jugular nodes (skull base; inferior body of the hyoid bone; stylohyoid muscle/vertical plane of the spinal accesory nerve; vertical plane of the spinal accesory nerve/lateral border of the sternocleidoid muscle)
**Level III**	Middle jugular group (inferior body of hyoid; inferior border of cricoid cartilage; lateral border of sternohyoid muscle; lateral border of sternocleidoid muscle or sensory branches of cervical plexus)
**Level IV**	Lower jugular group (inferior border of the cricoid cartilage; clavicle; lateral border of sternohyoid muscle; lateral border of sternocleidoid muscle or sensory branches of cervical plexus)
**Level VA and B**	Posterior triangle group (apex of convergence of sternocleidoid and trapezius muscle/lower border of cricoid cartilage; lower border of the cricoid cartilage/clavicle; posterior border of sternocleidoid muscle or sensory branches of cervical plexus; anterior border of trapezius muscle (7)
**Level VI**	Anterior compartment group (hyoid bone; suprasternal; common carotid artery; common carotid artery)

**Table 2:** Types of ND according to Robbins and Medina.

Types of neck dissection (ND)	Extent
Radical neck dissection (RND)	lymphatic structures and 1 or more of SAN, IJV, SCM
Selective neck dissection (SND)	defined levels of lymphatic structures without other structures
*Lateral/anterolateral/posterolateral*	
Modified radical neck dissection (MRND)	
*MRND I*	RND without SAN
*MRND II*	without SAN, IJV
*MRND III ("functional ND")*	without SAN, IJV, SCM
Extended neck dissection	RND with one or more lymphatic or other anatomical structures

Spinal accessory nerve: SAN, internal jugular veine: IJF and sternocleioid muscle: SCM; modified radical neck dissection: MRND, selective neck dissection: SND.

SND is the excision of one or more of the six cervical levels and is most commonly performed in OSCC as ND of levels I-III. *Medina* (1989) also proposed subclassifications for SND: lateral (levels II, III, IV), anterolateral (I-IV), suprahomohyoid (I-III), which preserve the SAN, IJF, SCM, and posterolateral. Extended ND includes additional resection of lymphatic structures in the parapharyngeal area or vessels such as the carotid artery. Recently, Cheng et al. (2020) described a predictive cutoff in 37 lymph nodes taken within their collective of 126 patients with OSCC (9). A neck dissection with fewer than 37 lymph nodes may show stage migration as part of underdiagnosis and has a lower survival rate.

Similarly, different and heterogeneous data regarding the reliability of MR and CT imaging in terms of staging examinations in OSCC patients are available thus far. Authors determine the sensitivity, for example, between 60 and 100% (10–12).

The aim of the present study was to evaluate the accuracy and predictive outcome with regard to the detection of cervical lymph node metastases in oral malignancies by comparing preoperative MRI and CT imaging (cN) with postoperative histopathological findings (pN) after a systematic bilateral neck dissection (ND).

## Materials and methods

An evaluation of all patients with histopathological evidence of OSCC and primary surgery in the Department of Oral and Maxillofacial Plastic Surgery, University Hospital Frankfurt, Germany, from 01 January 2014 to 31 December 2020 was performed. Observation period was until 31 October 2022. Patients were identified by evaluation of the internal database and confirmation by hand. Ethics approval was granted (03/2013; 40/18; 2021-76). The study has been registered in the German Clinical Trials Register (DRKS) Number 00016654.

The inclusion criteria were as follows: consecutive patients with histopathological proof of OSCC and operable primary manifestation, preoperative staging using MRI or CT, bilateral systematic ND, and histopathological examination of all lymph nodes labeled according to the *Robbins* (1991/2002) and Medina (1989) classifications.

Patients were excluded in cases of extraoral localization, primary nonsurgical intervention such as radiotherapy or a combination of radiotherapeutic and oncologic treatment.

All patients received MRI or CT of the head and neck region. Surgery consisted of primary resection of the tumor and structured bilateral systematic ND following oncology board meetings’ recommendations and a standardized approach: As patients with only unilateral ND were excluded, the criteria for bilateral ND were clinical signs of tumor positive nodes, primary tumor crossing the midline, intraoperative ipsilateral positive nodes proven by frozen sections or macroscopic invasion of the lymph nodes capsule, invasion of the base of the tongue, pharyngeal wall, tumor of the palate invading the palatal arch, tonsil and base of the tongue, according to the current German guideline (10).

ND was performed as anterolateral SND (I-IV) or MRND III or a combination of both, according to Medina (1989) (6, 8). In the case of an intraoperative hint or proof of ipsilateral lymph node metastasis, the SND was extended to an MRND III ipsilateral and a SND on the contralateral side has been added. If lymph node metastasis was found contralaterally, the contralateral SND was also extended to MRND III.

The dissected lymph nodes were explicitly labeled per level and sent separately – level by level – to the pathologist. Histopathological examinations were performed according to routine settings of the clinical pathology department.

The preoperative findings of lymph node involvement on MR and CT were correlated with the histopathological results after all tissue was sent for histopathological examination, especially for the presence and absence of lymph node involvement and the exact N-stage (N1,2,3).

Subgroups for patients undergoing MRI and CT were defined. Allocation to either MRI or CT as a local (cervical) presurgical staging method was performed according to the patient’s individual status and disposability of the modality. In addition, patients suspicious to infiltration of bony structures have been administered to CT due to the better contrast. Patients without suspicion of bony infiltration have been administered to MRI.

For the present study, the following parameters were recorded and evaluated: age, sex, anatomical localization of the tumor, TNM classification as T-stage, grading, lymph node involvement according to levels of the Robbins classification as depicted in preoperative radiological MRI or CT examination, and lymph node involvement according to histopathological findings.

Statistical analysis was performed using Excel and Prism GraphPad using a nonparametric Mann–Whitney test. P values <0.05 were considered to be significant.

The authors have read the Helsinki Declaration and have followed the guidelines in this investigation.

### CT protocol

Patients were examined on 192-slice third-generation dual-source CT (SOMATOM Force, Siemens Healthcare). The examinations were performed with the following scan parameters: tube voltage 120 kV with 120 reference mAs; pitch, 0.8; rotation time, 1.0 s; collimation, 192x0.6 mm. CT scans were acquired 70 sec after intravenous administration of 100 ml of nonionic iodinated contrast medium (Iopamidol, Imeron 400, Bracco, Konstanz, Germany) with a flow rate of 2 mL/sec in an expiratory breath hold and in the craniocaudal direction with the patient in the supine position.

### MRI protocol

MRI scans were acquired with a 3-T system (MAGNETOM PrismaFit, Siemens Healthineers) with a dedicated head and neck coil. Standard axial turbo inversion recovery magnitude (TIRM) (repetition time ms/echo time ms 3270/36; matrix size, 320 × 252; section thickness, 6 mm), coronal unenhanced T1-weighted turbo spin echo (repetition time ms/echo time ms, 718/9; matrix size, 320 × 288; section thickness, 4 mm), axial diffusion-weighted (repetition time ms/echo time ms, 3980/55; matrix size, 160 × 160; section thickness, 5 mm); axial unenhanced T1-weighted turbo spin echo (repetition time ms/echo time ms, 659/12; matrix size, 384 × 324; section thickness, 4 mm); axial T2-weighted turbo spin echo (repetition time ms/echo time ms, 7010/83; matrix size, 384 × 365; section thickness, 4 mm) sequences were acquired. In addition, axial enhanced T1-weighted multipoint Dixon with fat suppression (repetition time ms/echo time ms, 604/12; matrix size, 320 × 277; section thickness, 4 mm) and coronal enhanced T1-weighted turbo spin echo (repetition time ms/echo time ms, 718/9; matrix size, 320 × 288; section thickness, 4 mm) sequences were acquired. Contrast medium administration was performed with injection of 0.1 ml of gadobutrol per kilogram bodyweight gadobutrol at a flow rate of 2 mL/s using a power injector (Accutron MR; Medtron, Saarbrücken, Germany) followed by the application of 20 mL of saline solution at a rate of 2 mL/s.

The image evaluation was performed on a commercially available PACS workstation (Centricity 4.2, GE Healthcare, Dornstadt, Germany). Two different observers (1 resident of the radiology department, 1 senior staff member) analyzed the CT and MR series in consensus.

### Radiologists’ protocol

Two independent reviewers from the department of radiology screened MRI- and CT-datasets. In addition to this, specific images were reviewed at the oncology board meeting by a third reviewer.

The criteria for abnormal lymph node structure depends on size, configuration, homogeneity and borders of the lymph node according to the guideline by the particular institution. Lymph nodes were set more likely pathological with a diameter above 6 mm in the occipital, mastoidal, parotid, facial, retropharyngeal region; above 10 mm in submandibular, mental and clavicular region and above 12 mm in jugulodigastric area. Kidney shaped and/or lymph nodes with fatty hilus were more likely to be suspicious. Homogenous formation with central necrosis and homogeneity in an elevated number of lymph nodes were also more likely to be suspicious. Irregular border structures may hint at infiltration of surrounding structures and be a sign of an invasion through the capsule.

The verdict upon the node stage was based on an individual weighting of all criteria mentioned above.

### Pathologists’ protocol

Lymph nodes have been labeled by the surgeons according to the distinctive level and affected site. The samples were then sent to the department of pathology and were examined after routine protocols according to the current German guideline for histopathological examination in OSCC (11).

In the case of frozen sections, samples were examined macroscopically and subsequently examined microscopically. All signs of infiltration with different cells correlating with the primary OSCC were labeled tumor positive. All samples were prepared for fixation.

All lymph nodes were fixated in 4% formalin and stored for a 24 hour period. After macroscopic examination a microscopic histologic evaluation was carried out. Lymph nodes <1cm have been dissected in the middle into two pieces and examined microscopically. Lymph nodes >1cm have been cut in 4 mm slices and then examined microscopically. For each level the number of positive nodes and the size of the biggest positive lymph node was recorded. In case of infiltration of lymphatic vessels “L1” was noted. All extra tumoral nodes >10 mm distance from the primary tumor without lymphatic cells have been labeled as positive lymph nodes. All lymph node metastases and soft tissue metastases were handled according to the individual UICC-guideline per year.

## Results

The study reviewed a cohort of patients with primary tumor resection of oral squamous cell carcinoma (OSCC) between 2014 and 2020 with preoperative magnetic resonance imaging (MRI) or computed tomography (CT) as local staging examinations and correlated the findings with histopathological results of bilateral systematic neck dissection (ND). Each patient’s individual presurgical TNM classification based on radiological assessment was compared with the definite histopathological result leading to pTNM classification. Additionally, the exact individual cN-stage was correlated with pN.

### Patient collective

**Figure 1:**
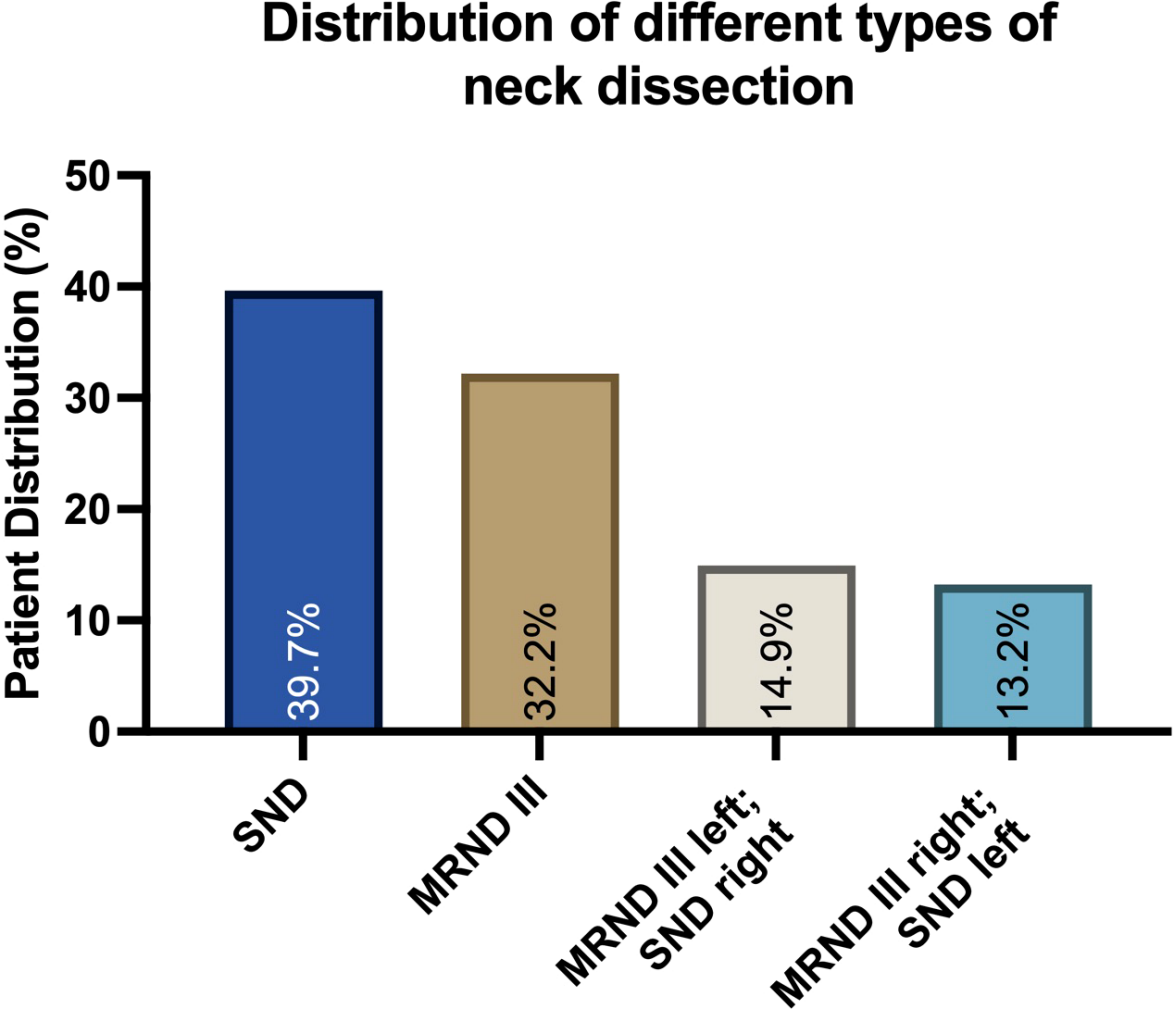
Distribution of different neck dissection types in a collective of 174 patients diagnosed with oral squamous cell carcinoma (OSCC). Data are represented as the percentage of the total, based on N=174 patients. Selective neck dissection (SND) and the anterolateral type were most frequent, followed by modified radical neck dissection (MRND) and a combination of both.

**Figure 2:**
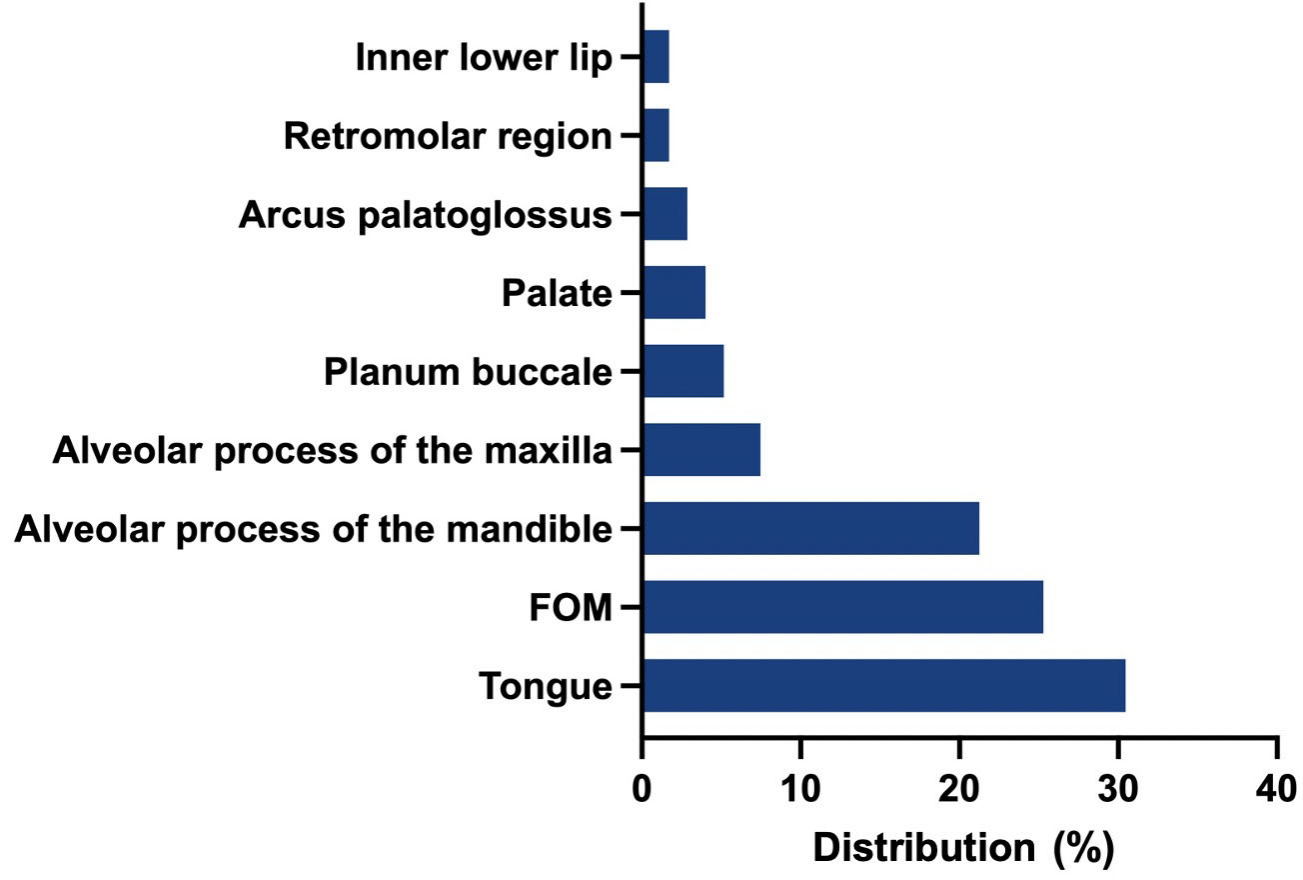
Frequency of primary localizations: Tongue was the most frequent localization (30.46%), followed by floor of mouth (FOM) with 25.29%, alveolar process of the mandible (21.26%), alveolar process of the maxilla (7.47%).

A total of 174 consecutive patients, almost equally female (89) and male (85), who fit the inclusion criteria, with a mean age of 64 years (SD 12.02; 30–92), were included in this study. Of these, 39.66% (N=69) received anterolateral-type selective neck dissections (SNDs) on both sides. Anterolateral selective ND comprises cervical levels I-IV without removal of nerves or vessels. A total of 32.18% (N=56) of the patients collectively received bilateral modified radical neck dissections (MRNDs). The extent of modified radical ND is levels I-V without any removal of nerves or vessels. A total of 28.16% (N=49) of all patients received a combination of SND on one side and MRND on the contralateral side (**Figure 1**). **Figure 2** depicts the primary localization of the tumor. Most frequent localization was the tongue with 30.46%, followed by floor of mouth (FOM) with 25.29%.

### Examination numbers and overview

Of 174 preoperative staging examinations, 78.74% (N=137) were conducted as MRI local staging examinations, and 21.26% (N=37) were conducted as CT examinations. The detailed cT status was as follows: T1 30.46% (N=53), T2 30.46% (N=53), T3 20.11% (N=35), and T4 18.97% (N=33) (**Table 3**). In 31.61%% (N=55) the tumor exceeded the midline and was present on both sides.

**Table 3:** cT status of 174 patients according to MRI, CT and overall groups.

T	Overall		MRI		CT	
	absolut	percent	absolut	percent	absolut	percent
**1**	53	30,46%	46	33,58%	7	18,92%
**2**	53	30,46%	41	29,93%	12	32,43%
**3**	35	20,11%	29	21,17%	6	16,22%
**4**	33	18,97%	21	15,33%	12	32,43%

Of all patients, 39% (N=68) showed lymph node involvement with at least one positive lymph node, 13.22% (N=23) were ranged as cN1, 12.07% (N=21) as cN2b, 6.90% (N=12) as cN2c, 0.57% (N=1) as cN3 and 6.32% (N=11) as cN3b (**Table 4**). In 8.62% (N=15) of all cases bilateral metastases were present.

**Table 4:** cN status of 174 patients according to MRI, CT and overall groups.

N	Overall		MRI		CT	
	absolut	percent	absolut	percent	absolut	percent
**0**	106	60,92%	87	63,50%	19	51,35%
**1**	23	13,22%	20	14,60%	3	8,11%
**2**	0	0,00%	0	0,00%	0	0,00%
**2b**	21	12,07%	15	10,95%	6	16,22%
**2c**	12	6,90%	6	4,38%	6	16,22%
**3**	1	0,57%	1	0,73%	0	0,00%
**3b**	11	6,32%	8	5,84%	3	8,11%
	174	100,00%	137	100,00%	37	100,00%

### Overall, MRI, and CT sensitivity and specificity

The overall sensitivity concerning the presence of any cervical lymph node involvement was 66%, and the specificity was 68%.

CT examinations alone showed a sensitivity of 67% and a specificity of 68%. In addition, the false-positive rate for CT examinations was 32%, and the false negative rate was 33%.

**Figure 3:**
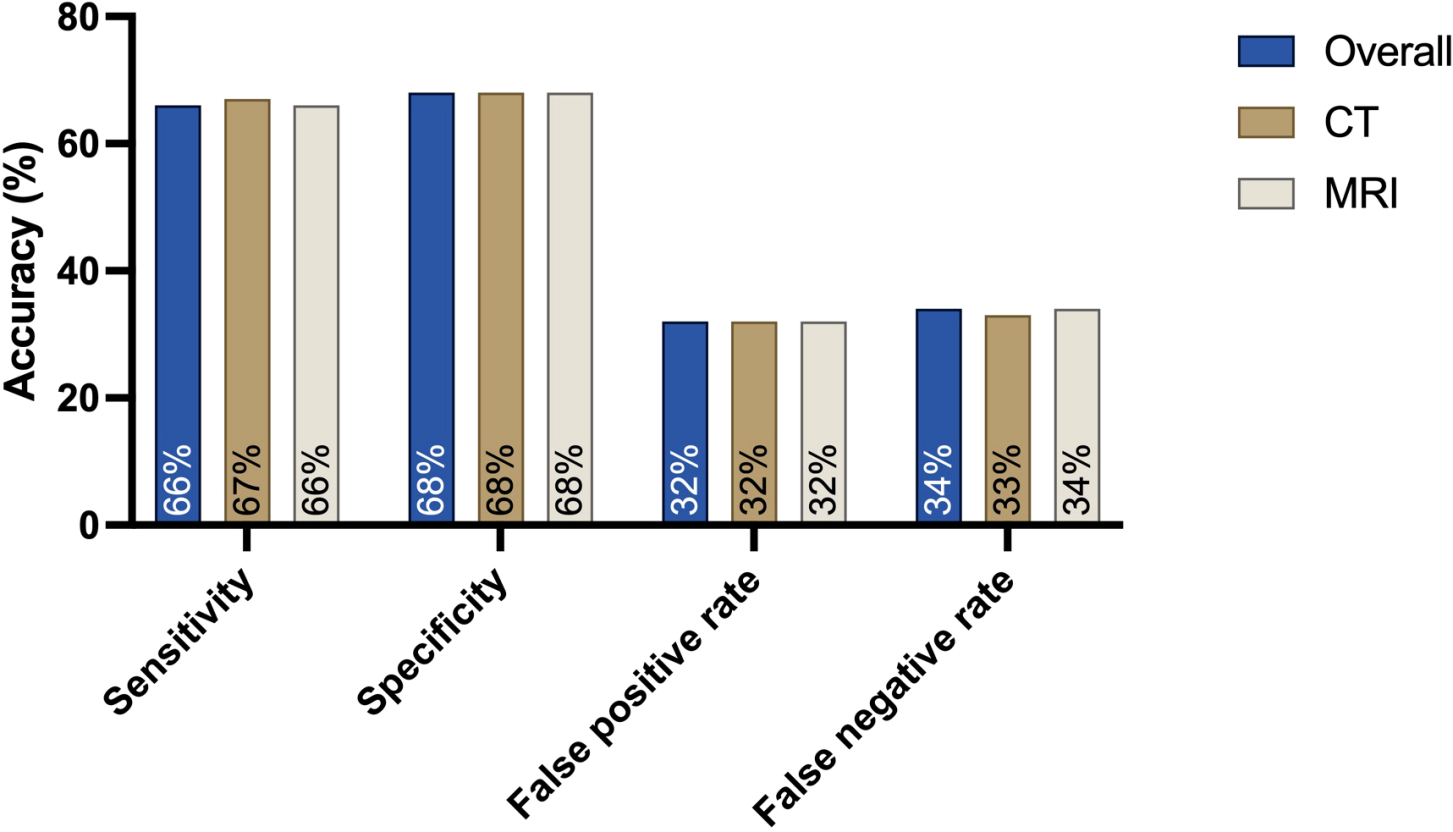
Sensitivity, specificity and false-positive and false-negative rates for the presence of any cervical lymph node metastasis when comparing the preoperative cN status based on MRI or CT findings with the postoperative histopathological results: CT is the leading modality, with a high sensitivity of 67%. The specificity for both MRI and CT is 68%.

For MRI alone, the sensitivity was 66% lower than that of CT, and the specificity was 68%. The false-positive rate was 32% higher than that of CT examinations, and the false-negative rate was 34% higher (**Figure 3**).

No significant difference was observed in correlating overall MRI or CT (p=0.76).

### Detailed correlation between staging examinations and histopathological findings (exact N status)

Overall preoperative staging examinations compared to the histopathological findings using MRI and CT revealed 52.87% identical information regarding lymph node involvement concerning the exact N-status. In contrast, in 47.13% of all cases, differences in the correlation of the exact N-stage occurred.

Concerning CT examinations, 56.76% of all cases of the clinical N status were identical to the histopathological findings pN (exact N-stage). In 21.62% of all cases, CT assumed a higher cN-stage than the histopathological pN findings, while in 21.62% of cases, a lower cN-stage was assumed.

MRI examinations were identical in 51.82% of all cases. In contrast to the CT-examinations in 27.00% of cases, MR assumed a higher cN-stage than the histopathological pN findings. In 21.17% of cases, cN-stage was assumed to be lower (**Table 5**; **Figure 3**). Both CT and MRI show a overestimation of lymph node involvement. The direct correlation indicates MRI as a method which is more likely to overestimate lymph node involvement in comparison to CT. No significant difference was observed in this correlation.

**Table 5:** Correlations between CT and MRI staging examinations (cN) and histopathological findings (pN) in the exact N-stage.

	lower	equal	higher
**Overall**	21,26%	52,87%	25,86%
**CT**	21,62%	56,76%	21,62%
**MRI**	21,17%	51,82%	27,01%

### Recurrence rate

**Figure 4:**
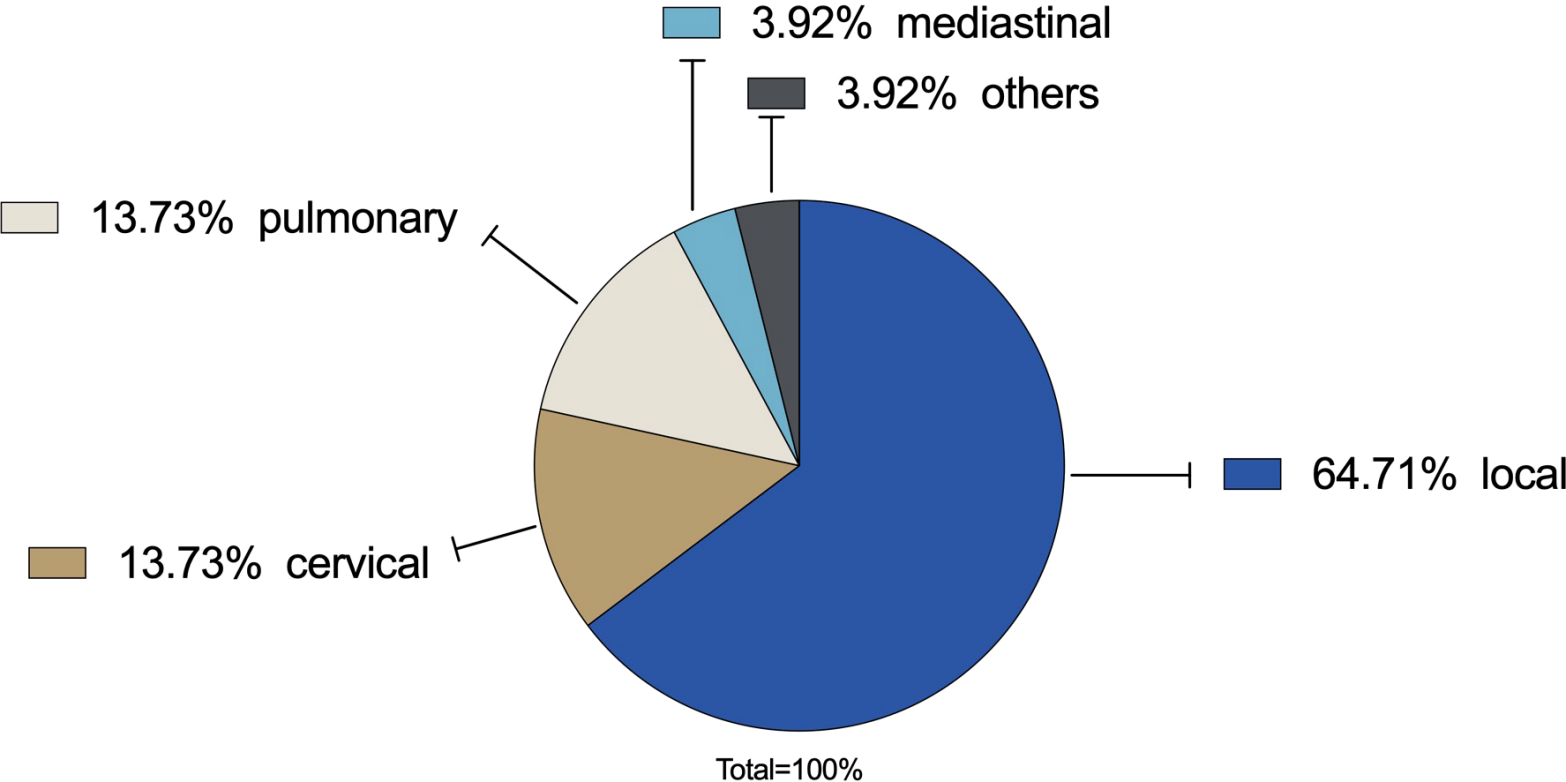
Recurrence rate: In 29.31% a relapse occurred. Local relapse was the most common type with 64.71% (N=33) followed by regional cervical lymph node metastases (13.73%, N=7) and distant pulmonary metastases (13.73%, N=7). Mediastinal metastases were found in 3.73% (N=2). Others were 3.73% (N=2).

Within the observation period until 31 October 2022, a relapse occurred in 29.31% (N=51) of all cases. The most common type was a local relapse in 64.71%. Both regional cervical and pulmonary metastases were 13.73% (N=7). In 3.73% metastases were found mediastinal. In 3.73% metastases were found in other regions (**Figure 4**).

## Discussion

The present study was designed to retrospectively investigate the accuracy and predictable outcome of presurgical staging examinations using magnetic resonance imaging (MRI) and computed tomography (CT) in patients with oral squamous cell carcinoma (OSCC) correlating with the histopathological results. This investigation is, to the best of our knowledge, the first utilizing a structured systematic bilateral approach based on the recommendation of an interdisciplinary oncology board meeting relying on preoperative MRI and CT.

The overall sensitivity of all MRI and CT examinations in the prediction of lymph node involvement was 66%. The overall accordance of all preoperative MRI and CT and the postoperative histopathological results concerning the exact N-stage (cN1/2/3 vs. pN1/2/3) was 52.87%.

CT alone showed a sensitivity of 67%. The CT examinations revealed higher accordance, as 56.76% were identical, and 21.26% assumed higher N-stage.

MRI alone had a sensitivity of 66%. The MRI examinations showed lower accordance, as the findings were identical in 51.82%, and in 27.00% of cases, a higher N-stage was supposed. This overestimation in the exact N-stage directly affects therapy decisions. In particular, in case of a false positive lymph node this might be the origin for the indication for bilateral neck dissection. In contrast, concerning CT examinations, 21.62% of cases were supposed to range in a lower N-stage, whereas MRI assumed a lower N-stage in 21.17% of cases.

The precondition for all retrospective evaluations was a systematic approach of bilateral ND, which was performed in this study as either anterolateral selective neck dissection (SND) or modified radical neck dissection (MRND) and a combination of both according to Medina et al. (8, 12). This structured systematic bilateral approach is needed for an analysis to represent the whole area of interest, which is scanned by MRI or CT: this comprises the primary cancer site and the locoregional pathway of metastases of OSCC of both sides of the neck, including Level 5 (6, 7). The decision for the type of ND and extent is routinely based on the preoperative findings, which lead to an oncologic board meeting proposal and thereby determine the extent of ND. This is of particular importance, as it directly affects mortality and morbidity plus the quality of life, which can be significantly poorer with a higher extent of ND (13, 14). In contrast, especially in early-stage T1 and T2 cancer, elective ND is more favorable to improve overall survival than a watchful wait strategy with therapeutic ND in cases of nodal relapse. The authors performed a 3-level ND for the elective group and a 5-level modified radical ND for the therapeutic surgery group (3). However, as the recent literature suggests a bilateral ND seems to be outdated as alternative diagnostic tools arise in order to prevent restrictions following ND (15–17).

In general, and recommended by the current German guideline, CT and MRI are equal in both staging the primary tumor and evaluating the cervical area (18, 19).

The results concerning the accuracy of CT and MRI are similar to values gathered 20 years ago, with sensitivities of 66% for CT and 64% for MRI (20). Restrictively, these data are not raised in comparison with a bilateral systematic approach underlying the present study. As some evaluations and the current German guideline see MRI as the more accurate method in evaluating soft tissue and lymph node involvement than CT, this study demonstrates that CT is the more favorable method (4, 19). Laimer et al., 20*20* investigated different modalities in head and neck presurgical staging and stated a 100% sensitivity for the combination of MRI plus CT and PET plus MRI regarding the highest sensitivity for CT alone with 95% (21). Stoeckli et al., 2012 found a sensitivity for CT of 86.9% in a cohort of not only OSCC but also head and neck squamous cell carcinoma (HNSCC) without involving MRI (22). Similar results of 92% sensitivity in patients with OSCC were described by Pandeshwar et al., 2013 focusing on a 1-cm lymph node size and central necrosis, adding that the combination with another method, such as ultrasound-guided fine needle aspiration biopsy, improves CT’s ability (23). Also, CT findings might be improved by adding ultrasound (24).

Other studies see MRI and CT as equal in detecting lymph node metastases (25, 26). However, 20 years of technical progress influencing MRI and leading to improved tissue contrast or functional imaging, the results in evaluating OSCC staging have not changed (20, 27). Laimer et al., 2020 observed a reduced sensitivity (85.7%) and specificity (75.6%) for MRI alone compared to CT. These findings are based on one-sided ND and are concordant with Yoon et al., 2009 (21, 26). Yoon et al. found a sensitivity of 77% for MRI and CT. In addition to inconsistent criteria for labeling lymph nodes as suspicious, another cause for differences in comparison can be moving artifacts, which may affect the outcome of MRI and are found especially in elderly people. These account for an increasing proportion of the affected patients.

Still, there are no standard criteria in imaging for labeling lymph nodes as tumor positive in OSCC. Different radiological criteria might predict a lymph node to be suspicious including size, homogeneity/heterogeneity, necrosis, perfusion defect or other tumor related risk factors (28–31). Artificial intelligence and deep learning may enhance the efficacy in detecting positive lymph nodes (32). Expanded and cut-off size or conglomeration of lymph nodes may be the cause for a false positive decision as they may also be a sign of general inflammation (33). Artifacts and inadequate contrast enhancement may also hint to misleading findings (34). Concomitant diseases like leukemia, benign lymphopatia and others may disguise a lymph node involvement of OSCC. Even diffusion-weighted imaging cannot reliably predict lymph node involvement (35).

Recently, some research favored MRI in measuring tumor thickness and local infiltration, thereby mentioning its value in evaluating soft tissue. However, this cannot imply predicting the existence of lymph node involvement (36, 37). MRI not only overestimates N status and the number of patients with lower N status but also has a higher false-negative rate. These findings make it difficult to prefer MRI as the modality of choice. In addition, both modalities CT and MRI overestimate the pN-stage and may lead to overtreatment by expanding not only the unilateral ND but also the bilateral ND.

In addition, cost- and time-effectiveness must be considered. For these reasons, undergoing just one modality should be the favorable method instead of a combination of numerous options. MRI of the head and neck remains a more expensive method than a CT scan (38). Nevertheless, certain diagnostic modalities are expensive and not covered by health injury providers for primary staging, as is the case in Germany. Additionally, exposure to radiation must be considered and may represent a risk while performing CT examinations.

Ultrasound was not considered in this study due to the aim of the study in comparing objectifiable imaging modalities such as MRI and CT. However, ultrasound remains an important tool for the standard clinical staging protocol with known interobserver variability. Although a dual-observer routine was obtained in the assessment of MRI and CT, the pairing of the two radiologists changed.

Addressing the current German guideline which is the basis for therapeutic strategies in patients with OSCC, the standard in local staging of OSCC is CT and MRI. CT, MRI and US are held to be equal in assessing the neck (10). However, US alone is less common due to lower specificity and examiner dependent accuracy (39). Furthermore, fine needle aspiration cytology is considered for a low specificity in a cN0 neck (40, 41). PET-CT is regarded as a low specificity tool (42). In fact, the current national guidelines discussion may rely on an outdated literature. However, the focus in a new discussion dealing with small tumor (T1/2) and/or clinical cN0 must consider the current literature concerning fine needle aspiration cytology or sentinel node biopsy. Flach et al. see no disadvantages in a “wait and scan”-policy within a 285 patients population in early stage cancer and cN0 neck (43). In occult metastases fine needle aspiration cytology has poor accuracy (44). In high risk patients freehand SPECT US might improve fine needle aspiration cytology (45). Also, PET-CT-scans may predict lymph node metastases in head and neck cancer verifying fine needle aspiration cytology findings (46). Mahieu et al. favor the role of sentinel lymph node biopsy as this method predicts better control of the contralateral clinically negative neck. For now, these findings apply in patients with lateralized or paramedian early-stage OSCC (17). In addition to it, sentinel lymph node biopsy may also reveal lymphatic drainage patterns (47).

In general, the population undergoing this retrospective evaluation is specific. Bilateral ND, especially in early stage cancer, need to be questioned and distinctive indications have to be set (15); alternative treatment strategies need to be discussed. Sentinel lymph node biopsy might be an alternative for early stage OSCC except floor of mouth localization (48). This also applies to occult lymph node metastases (49).

Even if there is a lack of accuracy (sensitivity, specificity), CT and MRI remain objective and reproducible methods for preoperative staging methods in OSCC patients. However, the decision for a bilateral ND should be based on distinctive criteria, which should not only rely on CT or MRI staging alone. Insecure cases of labeling a lymph node positive or not may demand further interventions like selective image-guided lymph node and sentinel lymph node biopsy or fine needle aspiration cytology. In particular, the routine treatment in adding a contralateral ND in case of an ipsilateral tumor positive node should be abandoned.

Thus, ongoing clinical research on OSCC metastasis pathways and the accuracy of staging examinations are strongly needed to standardize therapy regimens.

## Conclusion

Head and neck surgery is subject to constant change and depends on the continuous development of imaging modalities and therapeutic improvements. The present study analyzed a cohort of patients with OSCC and structured bilateral ND and correlated the presurgical findings with the postsurgical results of the latest MRI and CT imaging modalities. On this basis, a correlation between cN and the pN status was enabled. Although they lack sensitivity and specificity, MRI and CT are still within the modalities of choice concerning objectifiable presurgical staging in the diagnosis and therapy of OSCC. Bilateral ND should be questioned as there are different alternatives to depicting the neck status like fine needle aspiration cytology or sentinel lymph node biopsy. A routine expand to the contralateral side should be abandoned.

Further prospective research needs to be conducted on the stated preconditions to reassess the value of the aforenamed staging examinations and reduce the number of ND performed.

## Data availability statement

The raw data supporting the conclusions of this article will be made available by the authors, without undue reservation.

## Ethics statement

The studies involving human participants were reviewed and approved by Ethikkommission der Universitätsklinik Frankfurt, Theodor-Stern-Kai 7, 60590 Frankfurt. Written informed consent for participation was not required for this study in accordance with the national legislation and the institutional requirements.

## Author contributions

PT did conception and design of study, data acquisition and analysis, drafting of the article and reviewing final revision. AH did data acquisition and analysis, reviewing of the final article. IB, RS, TV participated in reviewing the final version. SG did conception, drafting and reviewing the final revision. All authors contributed to the article and approved the submitted version.
